# Draft Genome Sequence of *Pseudozyma brasiliensis* sp. nov. Strain GHG001, a High Producer of Endo-1,4-Xylanase Isolated from an Insect Pest of Sugarcane

**DOI:** 10.1128/genomeA.00920-13

**Published:** 2013-12-19

**Authors:** Juliana Velasco de Castro Oliveira, Renato Augusto Corrêa dos Santos, Thuanny A. Borges, Diego Mauricio Riaño-Pachón, Gustavo Henrique Goldman

**Affiliations:** Laboratório Nacional de Ciência e Tecnologia do Bioetanol (CTBE), Centro Nacional de Pesquisa em Energia e Materiais (CNPEM), Campinas, São Paulo, Brazila; Faculdade de Ciências Farmacêuticas de Ribeirão Preto, Universidade de São Paulo, São Paulo, Brazilb

## Abstract

Here, we present the nuclear and mitochondrial genome sequences of *Pseudozyma brasiliensis* sp. nov. strain GHG001. *P. brasiliensis* sp. nov. is the closest relative of *Pseudozyma vetiver*. *P. brasiliensis* sp. nov. is capable of growing on xylose or xylan as a sole carbon source and has great biotechnological potential.

## GENOME ANNOUNCEMENT

*Pseudozyma brasiliensis* sp. nov. strain GHG001 is a yeast-like species that belongs to the order *Ustilaginales*. This strain was isolated from the intestinal tract of a *Chrysomelidae* larva associated with sugarcane roots in plantations in Ribeirão Preto, São Paulo, Brazil, following an enrichment protocol for microorganisms that use xylose as a sole carbon source. Based on the phylogenetic analysis of the ribosomal operon, we suggest that GHG001 represents a novel species that we named *P. brasiliensis* sp. nov.; its closest relative is *Pseudozyma vetiver* (1). GHG001 can grow well in xylose or xylan as its sole carbon source, where it produces high levels of endo-1,4-xylanase from the glycoside hydrolase (GH) family GH11 (2), the members of which show higher specific activity than other eukaryotic xylanases. Xylanases are essential for breaking down hemicellulose of plant cell walls, and they are routinely added to enzyme cocktails for the saccharification of pretreated biomass and second-generation ethanol production. Xylanases have further commercial applications, such as in bread making, the manufacture of food, drinks, and textiles, bleaching of cellulose pulp, and xylitol production (3).

Here, we present the genome sequence of *P. brasiliensis* sp. nov. strain GHG001. This genome was sequenced on the Illumina HiSeq2000 system, generating 73,703,379 paired-end reads of 100 bp (insert size, 250 bp). The reads were preprocessed with the Fastx-Toolkit (http://hannonlab.cshl.edu/fastx_toolkit/). The genome size was estimated to be 22.09 Mbp based on *k*-mer count statistics (4), with an estimated coverage of 585×. The reads were randomly subsampled to a genome coverage of approximately 100×, and this subset was assembled using VelvetOptimiser and Velvet (5, 6). The remaining reads were used to extend the contigs and perform scaffolding using SSPACE Basic (7). The resulting assembly has 45 scaffolds, with a total length of 17,323,620 bp and an N_50_ of 720,612 bp. The average G+C content of the genome is 56.3%, which is similar to those of *Pseudozyma hubeiensis* SY62 (8) and *Pseudozyma antarctica* T-34 (9). We evaluated the completeness of the gene space using CEGMA (10), which revealed that the current assembly is 97.98% complete. The scaffolds were masked for repeats using RepeatMasker, and gene prediction was carried out with GeneMark (11), Augustus (12), and STAP (http://korflab.ucdavis.edu/software.html), using MAKER (13). Gene finders were trained with the CEGMA-produced gene models. A total of 5,768 protein-encoding genes were identified, which is similar to the gene content of other *Pseudozyma* spp. A search against the NCBI nr database revealed 2,361 protein-encoding genes with strong sequence similarity hits to proteins in that database, providing a preliminary landscape of the genomic and metabolic capabilities of *P. brasiliensis*. Ribosomal genes were identified with RNAmmer (14), and the rRNA operon repeats (small subunit [SSU], internal transcribed spacer 1 [ITS1], 5.8 S, ITS2, and long subunit [LSU]) were collapsed into a single scaffold (PSEUBRA_SCAF27). One hundred nineteen tRNA genes were identified with tRNAscan-SE version 1.3.1 (15). The scaffold PSEUBRA_SCAF26 contains the mitochondrial genome.

### Nucleotide sequence accession numbers.

This whole-genome shotgun project has been deposited at DDBJ/EMBL/GenBank under the accession no. AWXO00000000. The version described in this paper is version AWXO01000000.
